# Artificial intelligence to diagnosis distal radius fracture using biplane plain X-rays

**DOI:** 10.1186/s13018-021-02845-0

**Published:** 2021-11-25

**Authors:** Kunihiro Oka, Ryoya Shiode, Yuichi Yoshii, Hiroyuki Tanaka, Toru Iwahashi, Tsuyoshi Murase

**Affiliations:** 1 Department of Orthopaedic Surgery, Graduate School of Medicine, Osaka University, 2-2 Yamada-oka, Suita, Osaka 565-0871 Japan; 2 Ibaraki Medical Center, Department of Orthopaedic Surgery, Tokyo Medical University, 3-20-1 Chuo, Ami, Inashiki, Ibaraki 300-0395 Japan

**Keywords:** Artificial intelligence, Distal radius fracture, Small data, Computer-aided diagnosis

## Abstract

**Background:**

Although the automatic diagnosis of fractures using artificial intelligence (AI) has recently been reported to be more accurate than those by orthopedics specialists, big data with at least 1000 images or more are required for deep learning of the convolutional neural network (CNN) to improve diagnostic accuracy. The aim of this study was to develop an AI system capable of diagnosing distal radius fractures with high accuracy even when learning with relatively small data by learning to use bi-planar X-rays images.

**Methods:**

VGG16, a learned image recognition model, was used as the CNN. It was modified into a network with two output layers to identify the fractures in plain X-ray images. We augmented 369 plain X-ray anteroposterior images and 360 lateral images of distal radius fractures, as well as 129 anteroposterior images and 125 lateral images of normal wrists to conduct training and diagnostic tests. Similarly, diagnostic tests for fractures of the styloid process of the ulna were conducted using 189 plain X-ray anteroposterior images of fractures and 302 images of the normal styloid process. The distal radius fracture is determined by entering an anteroposterior image of the wrist for testing into the trained AI. If it identifies a fracture, it is diagnosed as the same. However, if the anteroposterior image is determined as normal, the lateral image of the same patient is entered. If a fracture is identified, the final diagnosis is fracture; if the lateral image is identified as normal, the final diagnosis is normal.

**Results:**

The diagnostic accuracy of distal radius fractures and fractures of the styloid process of the ulna were 98.0 ± 1.6% and 91.1 ± 2.5%, respectively. The areas under the receiver operating characteristic curve were 0.991 {*n* = 540; 95% confidence interval (CI), 0.984–0.999} and 0.956 (*n* = 450; 95% CI 0.938–0.973).

**Conclusions:**

Our method resulted in a good diagnostic rate, even when using a relatively small amount of data.

## Background

Distal radius fracture is an injury that occurs frequently among the elderly [1]. In the elderly, favorable wrist function can be maintained through appropriate immobilization with a cast or splint for minor displaced fractures. However, residual deformations can result in complications, such as wrist pain, a restriction in the range of wrist motion, and decrease in grip strength. Thus, appropriate initial diagnosis is essential [2, 3]. Artificial intelligence (AI) has been applied to various medical technologies. In medical image processing, techniques such as the automatic segmentation of each internal organ from computed tomography (CT) data [4] and diagnosis of lesions from skin images [5] are already in practical application. The highly accurate automatic diagnosis of fractures using plain X-rays by AI has also been investigated [6, 7]. One of the obstacles faced while developing high reliable AI for diagnosing disease is that big data of at least 1000 images must be used to train these AIs. Usually, orthopedic surgeons use two-direction plain X-ray images to diagnose fractures. In this study, we hypothesized that developing an AI system with a highly accurate diagnostic ability for distal radius fractures using plain two-direction X-rays is possible even when learning with relatively small data.

## Methods

This study was approved by the Ethical Review Board of our institution (approval number: 18137). We followed the principles of the Helsinki Declaration, revised in 2000. Each author has affirmed that the organization to which they belong has approved the protocols for humans. Each step performed in this study follows the ethical principles of research.

### Data collection

We obtained 369 plain X-ray anteroposterior images and 360 lateral images of distal radius fractures from 369 patients of over 18 years of age with distal radius fractures and 129 plain X-ray anteroposterior images and 125 lateral images of normal wrist of 129 people from three affiliated hospitals. Since not all patients with fractures had been examined with the plain X-rays of the normal wrist, the plain X-ray diagnosed as tenosynovitis (sprain of wrist without fractures) was included in the image data of normal side. Further, nine lateral fracture images were excluded because those were taken in an oblique position due to severe wrist pain. The image size of each plain X-ray image was 500 pixels × 625 pixels, and the pixel size was 0.4 mm × 0.4 mm. The clinical l diagnosis results by specialized orthopedic surgeons based on situations due to injuries, clinical findings, and imaging from clinical settings were used as the gold standard for fracture diagnosis. Images of the normal side of the patients with distal radius fracture without existing disorders, such as trauma, arthritis, or bone tumor, were used as images of normal wrists.

### Convolutional neural network

**Fig. 1:**
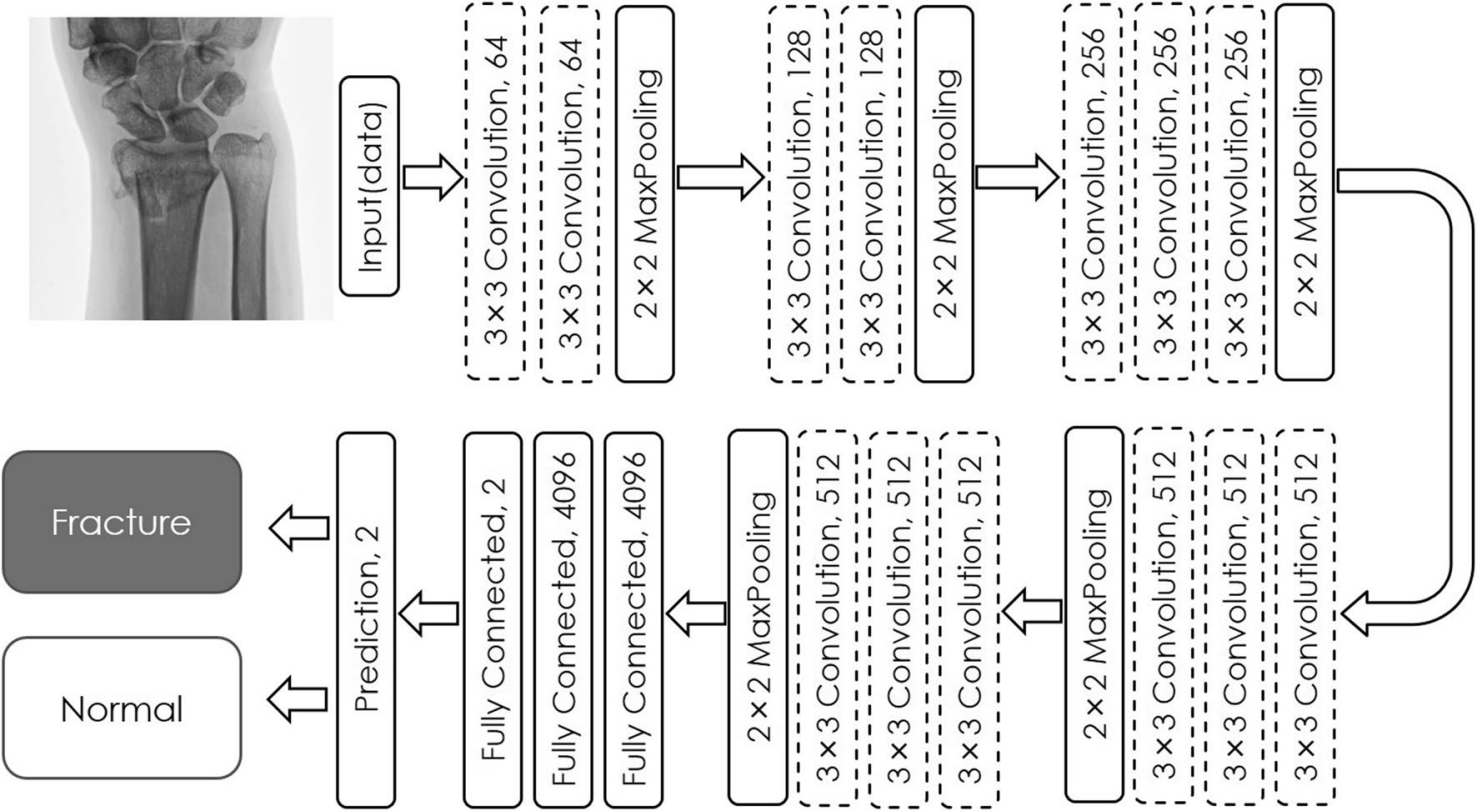
Schematic of the network structure. Input the images to determine whether there are fractures

VGG16, which is a model with already learned image recognition, was used as the CNN. [8] It is an open network that is already trained, comprises 16 layers, and identifies 1000 types of images. It was then modified into a network with two output layers to identify the existence of fractures in plain X-ray images (Fig. 1). To execute the CNN, we used Python as the programming language and Keras and TesorFlow as the software libraries.

### Dataset

**Fig. 2:**
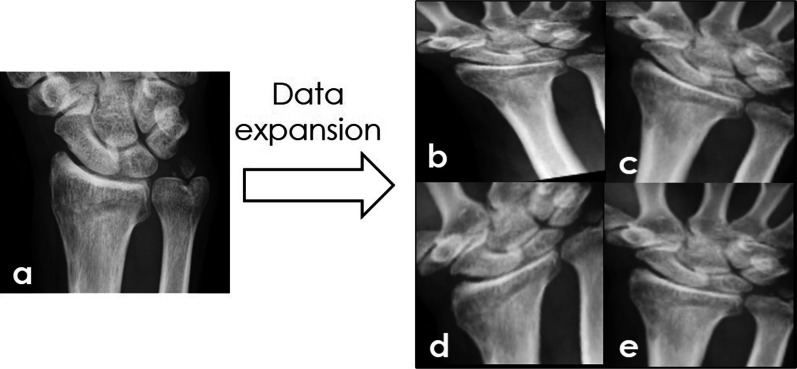
Data extension using affine transformation. **a** Original image. **b**–**e** Images that underwent arbitrary rotation, stretching, parallel translation, and shearing processing on the *xy* plane. The following equation was used for conversion. $x=sx*x\mathrm{cos}\theta -sy*\mathrm{ycos}\left(\theta +\mathrm{shear}\right)+t1$, $y=sx*x\mathrm{sin}\theta +sy*y\mathrm{cos}(\theta +shear)+t2$. *s:* Stretching (range ${e}^{-log1.3}-1.3$), *θ* Rotation angle (range − 25° to 25°), *t*1, 2: Parallel translation (range − 50 pixels to 50 pixels), *shear*: Shearing (range − 20° to 20°)

We used digital imaging and communications in medicine (DICOM) data of plain X-ray of a wrist as the original file with 16 bits per pixel. The images of the left wrist were reversed to enable every X-ray image to show a right wrist. The 729 fracture images (369 anteroposterior and 360 lateral images) and 254 normal wrist images (129 anteroposterior and 125 lateral images) were randomly selected to produce the following three patterns of datasets (A, B, C) to consider the effect of data selection on results. The dataset for training contains 569 fracture images (299 anteroposterior and 270 lateral images) and 174 normal wrist images (91 anteroposterior and 83 lateral images). In addition, the dataset for validation contains 80 fracture images (30 anteroposterior and 50 lateral images) and 40 normal wrist images (18 anteroposterior and 22 lateral images). The dataset for tests contains 80 fracture images (40 anteroposterior and 40 lateral images) and 40 normal wrist images (20 anteroposterior and 20 lateral images). These datasets are presented in Table 1. To increase the learning data, data were augmented by adding stretching, rotation, shearing, and parallel translation to the original images using affine transformation {$$\left(\begin{array}{c}{x}^{^{\prime}}\\ {y}^{^{\prime}}\end{array}\right)=\left(\begin{array}{cc}a& b\\ c& d\end{array}\right)\left(\begin{array}{c}x\\ y\end{array}\right)+\left(\begin{array}{c}tx\\ ty\end{array}\right)$$, *x* and *y* are the original coordinates; *x*′ and *y*′ are the coordinates after the conversion; *tx* and *ty* are the parallel translations}. These produced data with 3245 fracture images and 3210 normal wrist images are used for training and validation (Fig. 2). To adjust VGG16 for learning, conversion into 224 pixels × 224-pixels image size was conducted using a floating point of 32 bits per pixel. Moreover, to identify the fractures of the styloid process of the ulna, which often accompanies distal radius fractures, 189 anteroposterior images of the fractures of the styloid process of ulna and 302 images of the styloid process of the ulna without fractures were augmented into 845 and 1360 images, respectively, and three patterns (A, B, C) of datasets were prepared using the same approach (Table 1).

**Table 1:** Dataset

	Clinical diagnosis	Direction	Training*	Validation*	Test^†^	Total*
Distal radius fracture (*n*)	Fracture	AP	1495 (299)	150 (30)	40	1645 (369)
		LT	1350 (270)	250 (50)	40	1600 (360)
	Normal	AP	1365 (91)	270 (18)	20	1635 (129)
		LT	1245 (83)	330 (22)	20	1575 (125)
Ulnar styloid fracture (*n*)	Fracture	AP	745 (149)	100 (20)	20	845 (189)
	Normal	AP	1210 (242)	150 (30)	30	1360 (302)

^*^Number of augmented data (original data), ^†^number of original data

### Training

Learning was conducted by entering the training dataset after image augmentation into the network. Subsequently, the validation dataset was used for validation, and weighting was conducted to enable the output to approximate the correct answer using the back-propagation ($w\leftarrow w-\eta \frac{\partial E}{\partial w}, b\leftarrow b-\eta \frac{\partial E}{\partial b}$). Three learnings of approximately 40 epochs were conducted with each dataset for three patterns (A, B, C) because even if the same data set was used for training, there would be slight differences in the test results. Thus, nine learnings were conducted. The parameter of the epoch number where the diagnostic rate peaked during each learning was adopted, and three diagnostic tests for each pattern (a total of nine tests) were conducted. The nonaugmented original image data were used for the diagnostic tests of distal radius fractures and fractures of the styloid process of the ulna.

### Test and diagnosis

**Fig. 3:**
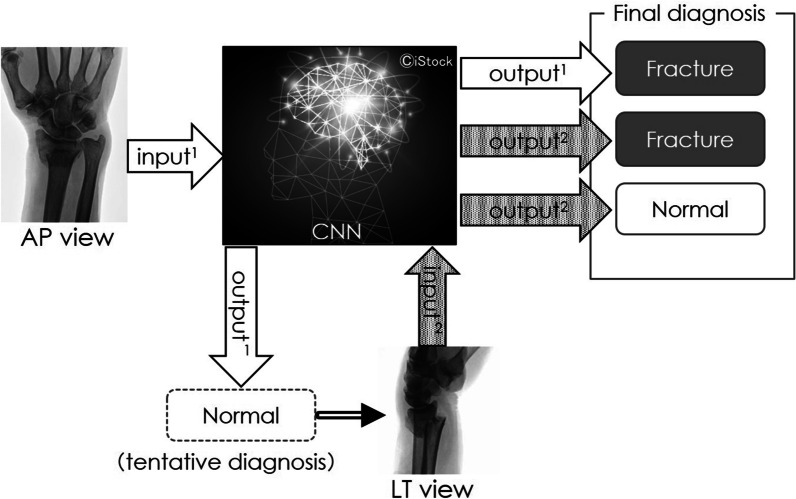
Distal radius fracture identification procedure. Input a plain X-ray anteroposterior image and if it identifies a fracture, it is diagnosed as a fracture. If it is determined to be normal, a lateral image of the same patient is input to make final determination regarding whether it is normal or a fracture

**Fig. 4:**
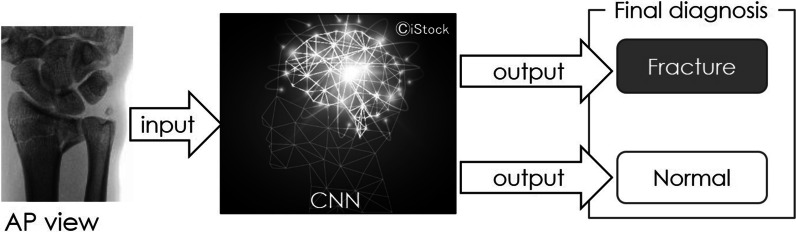
Identification procedure for the fractures of the styloid process of the ulna. Input a plain X-ray anteroposterior image to diagnose whether it is a fracture or normal

The method to determine a distal radius fracture involved entering an anteroposterior image of the wrist for testing into the trained AI. If it identifies a fracture, it is diagnosed as the same. When the anteroposterior image is determined as normal, the lateral image of the same patient is entered. If a fracture is identified in the lateral image, the final diagnosis is fracture; if the lateral image is identified as normal, final diagnosis is normal (Fig. 3). As the fracture in the lateral image of a styloid process of the ulna overlaps with the distal radius, making it difficult to identify, its identification during diagnosis is conducted only with the anteroposterior images (Fig. 4).

### Assessment

For the evaluation of the developed AI, we evaluated its diagnosis accuracy, sensitivity, and specificity using 40 images of distal radius fractures, 20 images of normal wrists, 20 images of fractures of the styloid process of the ulna, and 30 images of a normal styloid process of the ulna. We used the receiver operating characteristic (ROC) curve and area under curve (AUC) to evaluate the diagnostic ability. The time required for the process, which includes the extraction of the input data from the simple X-ray DICOM data and the diagnosis by the AI, was evaluated.

## Results

**Fig. 5:**
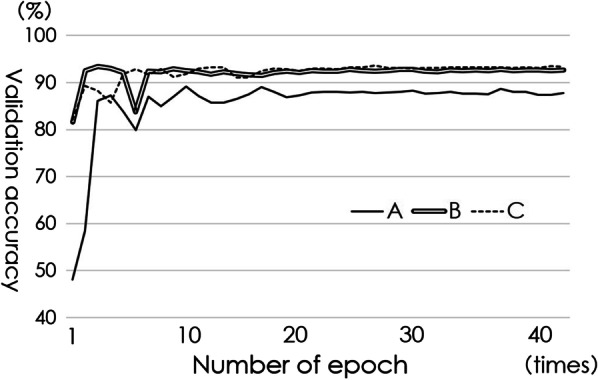
Epoch numbers of learning using datasets A, B, and C, as well as validation accuracy

**Fig. 6:**
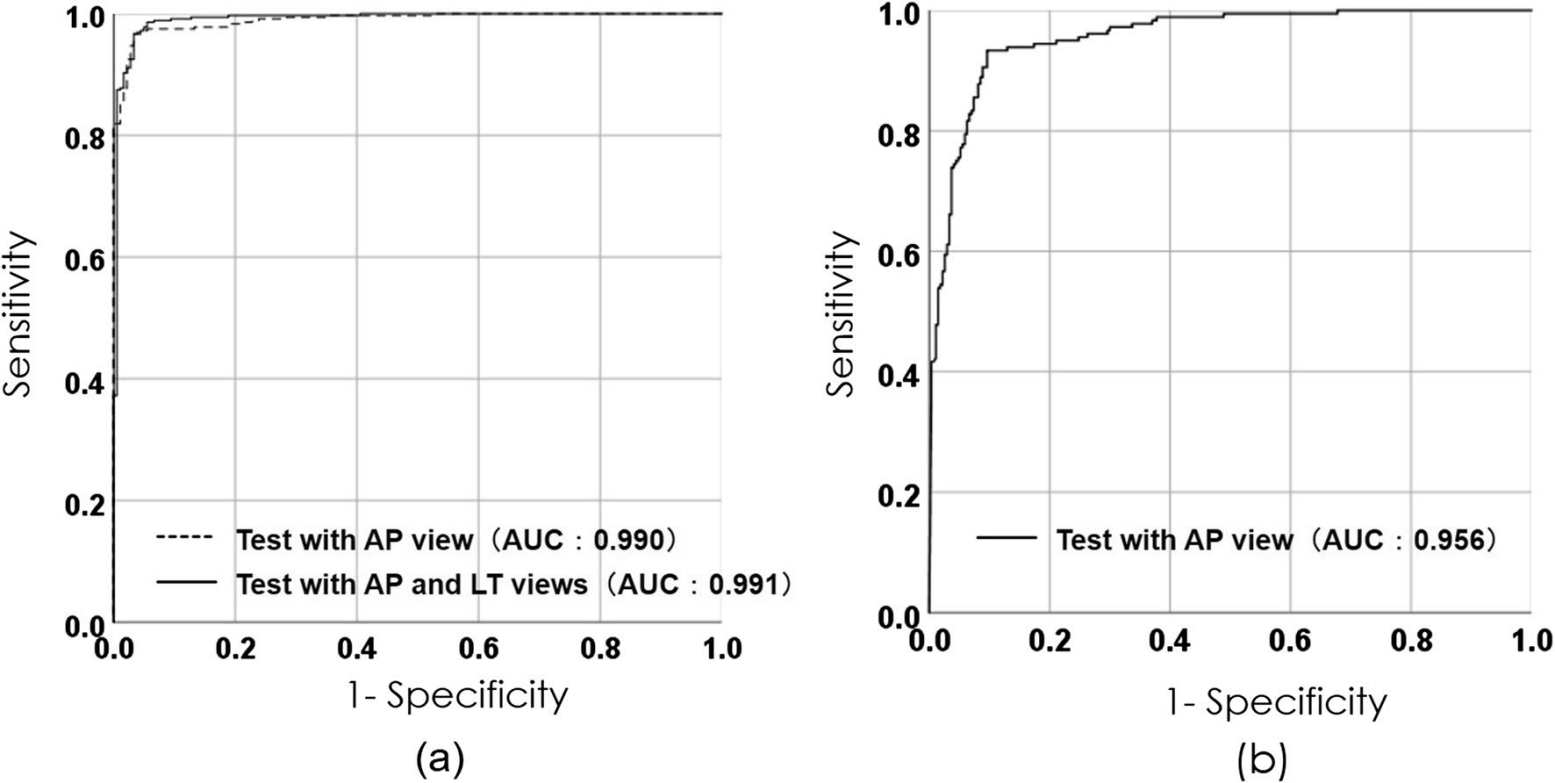
**a** ROC curve of the diagnostic tests of distal radius fractures. AUC of diagnoses using plain X-ray anteroposterior images is 0.990, and AUC of fracture diagnoses using both the anteroposterior and lateral images is 0.991. **b** ROC of diagnostic tests for fractures of the styloid process of ulna. AUC of fracture diagnoses using the plain X-ray anteroposterior images is 0.956

The number of epochs with the accuracy peak for validation in the diagnosis of distal radius fracture was 9, 3, and 25 for patterns A, B, and C, respectively. Their respective diagnostic accuracies were 89.2%, 93.5%, and 93.6%. The diagnostic accuracy after this point mostly plateaued (Fig. 5). The diagnostic accuracy of the anteroposterior images of distal radius fractures in the respective optimal epoch number of patterns A, B, and C was 95.7 ± 1.7%; the sensitivity and specificity were 95.0 ± 3.1% and 97.2 ± 2.6%, respectively. When the lateral images were input, the diagnostic accuracy increased to 98.0 ± 1.6%; the sensitivity and specificity were 98.6 ± 1.8% and 96.7% ± 3.5, respectively. The diagnostic accuracy of fractures of the styloid process of the ulna in the respective optimal epoch numbers of three patterns was 91.1 ± 2.5%; the sensitivity and specificity were 92.2 ± 5.7% and 90.4 ± 3.9%, respectively. Figure 6a shows the ROC of the diagnostic tests of distal radius fractures. The AUC of the diagnostic test using only the anteroposterior images was 0.990 {*n* = 540; 95% confidence interval (CI), 0.984–0.996} and that of the test using both anteroposterior and lateral images was 0.991(*n* = 540; 95% CI 0.984–0.999). Figure 6b shows the ROC of diagnostic tests of the styloid process fracture of the ulna. The AUC of the diagnostic of fractures of the styloid process of the ulna using anteroposterior images was 0.956 (*n* = 450; 95% CI 0.938–0.973). The time required for image conversion from the DICOM data and diagnosis by AI was approximately 30 s.

## Discussion

The initial diagnosis of trauma, such as distal radius fractures, is often performed by medical interns or emergency room doctors. It is possible to prevent the displacement of a fracture by appropriately diagnosing and performing immobilization with a cast or splint. Thus, initial diagnosis and treatment are essential [9]. The image recognition accuracy using AI is superior than the one by humans, and it has been applied to various fields [10]. The AI-based automatic fracture diagnosis system enables speedy diagnostic support of traumas and the initiation of treatment based on the diagnosis, which can be expected to improve the total treatment. Reports on existing bone image diagnosis using AI include a program that diagnoses bone age by learning the shape of the epiphyseal nucleus and bone maturity from the data of more than 10,000 plain X-ray images of children’s hands [11]. The accuracy of diagnosing bone age is 90.4% within 1 year and 98.1% within 2 years. Although a physician would require a few minutes to diagnose the bone age, AI can do so in less than 2 s. There is a report on the fracture diagnostic program that learned the location, plain X-ray direction, identification of fractures, and identification of left and right using data from more than 250,000 plain X-ray images of hand and foot [7]. Its accuracy in determining the location, direction, and left and right is more than 90%. The accuracy in identifying fractures is 83%, which is the same standard as specialized orthopedic surgeons. This result is expected to ensure clinical application. Regarding distal radius fractures, an AI that completed learning using approximately 35,000 plain X-ray images of wrists was approved by the United States Food and Drug Administration in 2018 [6]. It is a program that first diagnoses whether there is a fracture or not. If it identifies a fracture, it displays the location of the fracture in a heat map according to the trustworthiness of the diagnosis. The AUC of its fracture identification ability is 0.975, which is highly accurate. It has been reported that the support from this program reduced diagnostic error rate among emergency room doctors by 47%. Our developed program used less data for its learning than other reports, which was between 1/100 and 1/1000. However, with diagnostic accuracy of distal radius fractures at 98.0 ± 1.6% and AUC at 0.991, we obtained similar results with the same standard or even better than the previous reports. It is inferred that a good diagnostic rate was obtained, despite using a relatively small amount of data, due to the employment of the trained VGG16 model as the base, increasing the learning data up to the optimal quantity through image augmentation, and because the diagnosis was conducted in two stages using anteroposterior and lateral images, as in the case with the diagnosis by clinicians. It displayed good sensitivity at 98.6 ± 1.8%; hence, it is considered to be useful as a screening tool for initial diagnosis. However, although the diagnostic rate of fracture of the styloid process of the ulna was lower than that of distal radius fractures at 91.1 ± 2.5%, it is inferred that its diagnostic accuracy will improve if the number of datasets is increased to the same level as that used for distal radius fractures.

There are several limitations to this study. First, we used a small amount of learning data. It is not shown whether the same result can be obtained if the same number of data, other than the one used for this study, is used for learning. Second, because clinical diagnoses by orthopedic surgeons from clinical sites were used as the gold standard for correct identification of fractures, imaging tests, such as CTs, were not performed for all examples. However, the data included in this study were diagnosed as fracture by confirming the callus formation in the subsequent course even if fractures were diagnosed without CT. Third, it did not examine minute fractures that can be discovered using CT and other image detections or old fractures such as distal radius malunions and ulnar styloid nonunion. It is necessary to conduct further learning using more data, considering the diagnosis of fractures with hardly any displacement and old fractures. Finally, the data used in this study are from adults over 18 years, whose epiphyseal lines are already closed. Thus, AI cannot identify fractures for children’s bones, where epiphyseal lines still remain. A new network must be constructed for fractures in children.

Image diagnosis using AI is expected to improve significantly in the future. However, although imaging is one of the important examinations in the diagnosis of disease, comprehensive assessment of other clinical examination results, such as clinical histories, physical findings, and blood tests, is essential. In addition, it is to be remembered that image diagnosis using AI is only a supplementary diagnosis. Cohort studies and large randomized controlled trials are also needed to increase the reliability of AI-based diagnostics and predictions of injury in the field of orthopedics [12, 13].

## Conclusion

In conclusion, our method resulted in a good diagnostic rate even when using a relatively small amount of data. The image diagnostic technologies using AI are speedy. They are highly applicable technologies that can be used in the diagnosis of every disease appearing on plain X-ray, CT, or magnetic resonance imaging (MRI). Thus, further application in clinical sites is expected.

## Data Availability

The datasets used and analyzed during the current study are available from the corresponding author on reasonable request.

## Abbreviations

AI: Artificial intelligence
CNN: Convolutional neural network
CI: Confidence interval
CT: Computed tomography
DICOM: Digital imaging and communications in medicine
ROC: Receiver operating characteristic
AUC: Area under curve
MRI: Magnetic resonance imaging
